# Quantum secure metrology for network sensing-based applications

**DOI:** 10.1038/s41598-023-38802-6

**Published:** 2023-07-19

**Authors:** Muhammad Talha Rahim, Awais Khan, Uman Khalid, Junaid ur Rehman, Haejoon Jung, Hyundong Shin

**Affiliations:** 1 Department of Electronics and Information Convergence Engineering, Kyung Hee University, Yongin, Republic of Korea; 2 Interdisciplinary Centre for Security, Reliability and Trust (SnT), University of Luxembourg, 1855 Luxembourg, Luxembourg

**Keywords:** Quantum information, Quantum metrology

## Abstract

Quantum secure metrology protocols harness quantum effects to probe remote systems with enhanced precision and security. Traditional QSM protocols require multi-partite entanglement, which limits its near-term implementation due to technological constraints. This paper proposes a QSM scheme that employs Bell pairs to provide unconditional security while offering precision scaling beyond the standard quantum limit. We provide a detailed comparative performance analysis of our proposal under multiple attacks. We found that the employed controlled encoding strategy is far better than the parallel encoding of multi-partite entangled states with regard to the secrecy of the parameter. We also identify and characterize an intrinsic trade-off relationship between the maximum achievable precision and security under the limited availability of resources. The dynamic scalability of the proposed protocol makes it suitable for large-scale network sensing scenarios.

## Introduction

High-resolution sensors are pivotal for applications ranging from autonomous transportation to smart health-care systems^1^. Such sensing networks carry sensitive information that must be transmitted securely. However, when operated using classical components, these networks suffer from two significant challenges namely, statistical errors which limit the achievable precision and the rise of computational resources that may compromise information security. Contrarily, this incapacity is circumvented by incorporating quantum resources which enhances the achievable accuracy and provide unconditional security^2–13^.

Quantum metrology protocols operated over quantum sensing networks typically consist of three fundamental steps: (i) the creation of an appropriate probe state, (ii) its evolution in the system of interest described by a physical parameter $\phi$, and (iii) the measurement of the encoded state^5–9,14^. The encoded state is measured by employing suitable measurement settings, and the measurement results are processed to formulate an estimate of the parameter. The parameter $\phi$, which describes the unknown system of interest, is induced by the action of the Hamiltonian generator *H*. The evolution of the state $\rho _o$ to $\rho _\phi$ can be described by the unitary map $U_\phi = e^{-i\phi H}$. Using a set of measurement operators $M_x$, the probability distribution post measurement is obtained by utilizing the Born rule $P(x|\phi ) = \textrm{tr} [M_x\rho _\phi ]$. The associated quantum Fisher information (QFI) is

1 $$\begin{aligned} F_\phi = \textrm{tr} [\rho _\phi L^2], \end{aligned}$$

where *L* is the symmetric logarithmic derivative^6,7^. The Fisher information is directly related to the displacement of the probe state caused by even slight fluctuations of the parameter. An appropriate measure of the accuracy of our estimate is the units-corrected mean squared error of the estimate^14,15^

2 $$\begin{aligned} \delta ^2 \phi = \Bigg \langle \Bigg [ \frac{{\hat{\phi }}}{|\frac{\partial }{\partial \phi } \langle {\hat{\phi }} \rangle |} -\phi \Bigg ]\Bigg \rangle . \end{aligned}$$

Using the theory of error propagation^7^, we reduce this problem to measurements on an observable *O* which is derived after *v* repetitions of the experiment as

3 $$\begin{aligned} \delta ^2 \phi = \frac{\displaystyle \langle \;(\Delta O)^2\rangle }{v \displaystyle \bigg | \frac{\partial \langle O \rangle }{\partial \phi } \bigg |^2}. \end{aligned}$$

Conventionally, quantum metrology leverages quantum entanglement to surpass the standard quantum limit (SQL) and achieve measurement precision up to the Heisenberg limit (HL)^5–7,14^. In addition, the quantum states have a non-deterministic nature leading to uncertainties in their characterization. Therefore, they are employed in quantum cryptography protocols to provide information-theoretic security^2,3^. The integration of aforementioned concepts is fundamental to quantum secure metrology (QSM) protocols, through which we can sense a remote system with precision beyond the SQL while ensuring the transmission of the sensing parameter with unconditional security^10–13,16–18^.

Efficient quantum metrology protocols for network sensing-based applications require robust entanglement generation and distribution. In the noisy intermediate-scale quantum (NISQ)^19^ era, constraints on quantum hardware limit the practical applicability of such protocols. Most of the proposed QSM protocols utilize multi-partite entangled states^10,16–18^. However, generating genuine *N*-partite entanglement is based on probabilistic or indirect methods^20,21^. As a consequence, it significantly increases the required resources to faithfully establish genuine multipartite entanglement over quantum sensing networks. A suitable alternative is a bipartite entangled state such as the Bell state^22^, whose generation is relatively trivial. They can be expressed as one of the four orthogonal states:

$$\begin{aligned} \left| \Phi ^+ \right\rangle&= \frac{\left| 00 \right\rangle + \left| 11 \right\rangle }{\sqrt{2}}\\ \left| \Phi ^- \right\rangle&= \frac{\left| 00 \right\rangle - \left| 11 \right\rangle }{\sqrt{2}}\\ \left| \Psi ^+ \right\rangle&= \frac{\left| 01 \right\rangle + \left| 10 \right\rangle }{\sqrt{2}}\\ \left| \Psi ^- \right\rangle&= \frac{\left| 01 \right\rangle - \left| 10 \right\rangle }{\sqrt{2}}. \end{aligned}$$

Recent proposals have pointed towards their deterministic creation^23–25^, which lead to achieving relatively higher entanglement generation rates. Furthermore, increasing the number of entangled particles subjects the state to superdecoherence^26^, thereby compromising its distribution by reducing the transfer fidelity^27^. Similarly, distribution mechanism of Bell states is relatively more robust and less expensive than multi-partite states. For practical implementation in the near future, QSM protocols have to be designed employing fewer entangled particles, an idea that our proposed protocol embodies.

In this article, we propose a QSM protocol that involves two parties, Alice (source) and Bob (encoder), who perform QSM using Bell pairs. We utilize a controlled encoding strategy, eliminating the need for arbitrary states and additional gate operations to conceal the probe states from an eavesdropper. The security of our protocol is based on Bell’s theorem^3,28,29^ and does not require the need for a pre-shared key. We supplement our protocol with a security analysis for multiple attacks and prove its robustness against them. As we assume a resource-constrained setting, we characterize the trade-off relationship between the protocol’s maximum achievable precision and security. We show this trade-off mathematically and provide a bound on the allocation of resources to guarantee information security while providing a quantum advantage in precision. Such a case is relevant to real-world scenarios where the available resources are finite. Finally, we show that the proposed protocol may easily be extended to a multi-party scenario.

**Figure 1:**
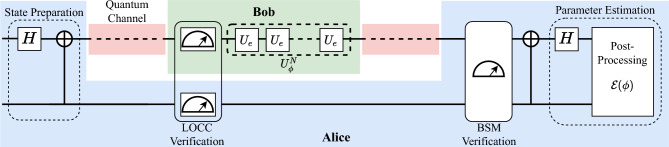
Implementation of the QSM protocol. Alice prepares the probe state and sends it to Bob through a quantum channel. After verification, Bob performs the sequential encoding process and returns it to Alice who performs the secure parameter estimation.

## Methods

In this section, we will provide the quantum secure metrology (QSM) protocol along with a comprehensive performance analysis.

### Quantum secure metrology (QSM)

The QSM protocol allows participants to estimate the parameter with enhanced precision while also ensuring its security. Firstly, we will discuss the system model of this protocol followed with detailed steps involved in its implementation.

#### System model

Our model consists of two participants, Alice and Bob, as illustrated in Fig. 1. They are connected via a quantum channel and communicate publicly through an authenticated classical channel (similar to quantum key distribution protocols). Both participants can perform local operations and classical communication (LOCC). Specifically, Alice is in charge of probe state preparation and parameter estimation. Herein, the Bell state is idealized as a metrological probe. Bob performs sequential parameter encoding on these probe states. In the following, we present our protocol.

#### Protocol

1. **Probe state preparation and distribution:** First stage of the protocol contains the following steps:
  - Alice generates $\ell$ Bell pairs of the form
    4 $$\begin{aligned} \left| \Phi _{AB} \right\rangle = \frac{\left| 0_A0_B \right\rangle + \left| 1_A 1_B \right\rangle }{\sqrt{2}}. \end{aligned}$$
  - She prepares two ordered sequences, $S_j$, $j \in \{A, B\}$. $S_j$ contains the $q_{j}^i$ particles of the $\ell$ Bell pairs, where $i \in \{ 1, 2, \cdots , \ell \}$.
  - She keeps the sequence $S_A$ for herself and sends the sequence $S_B$ to Bob via the quantum channel.
2. **Verification:** After receiving the sequence $S_B$, Bob verifies the Bell pairs by the following method:
  - Bob randomly selects the particles from the sequence $S_B$ with probability $\left( 1 - P_E\right) /2$ . He also randomly selects the measuring basis either $\sigma _x$ or $\sigma _z$ for each particle.
  - Then he announces the selected basis and particle location to Alice via the classical authenticated channel.
  - Both parties measure their particles and Alice announces her measurement results.
  - Bob compares the measurement results. If he finds errors in the correlation, he will abort the protocol; otherwise, he will continue to the next step.
3. **Sequential encoding:** After the verification stage, Bob discards the particles that were used for verification. Bob performs the sequential encoding on the $q_{B}^i$ according to the following rule:
  $$\begin{aligned} U_{B}^i = {\left\{ \begin{array}{ll} U_{E}^{N}, \, \mathrm { with \, probability \,\,} P_E,\\ U_{I}, \, \mathrm { with \, probability \, \,} \frac{1 - P_E}{2}.\end{array}\right. } \end{aligned}$$
  Here,
  $$\begin{aligned} U_E= & {} \left| 0 \right\rangle \left\langle 0 \right| + e^{\iota \phi } \left| 1 \right\rangle \left\langle 1 \right| \; \; \;\text {and,} \\ U_I= & {} \left| 0 \right\rangle \left\langle 0 \right| + \left| 1 \right\rangle \left\langle 1 \right| \end{aligned}$$
  where $\phi$ is the encoded parameter. After the encoding process, Bob sends the particles back to Alice.
4. **Secure parameter estimation:**
  - Bob announced the location of the particle selected for security of the quantum channel.
  - Alice measure these probe states in the Bell basis and estimates the error rate. If she finds an error in the probe states, she will abort the protocol; otherwise, she will continue to the next step.
  - Alice performs the parameter estimation by measuring the encoded probe states in the Bell basis and calculates the quantum Crámer-Rao bound.
    5 $$\begin{aligned} \delta ^{2}\phi \ge \frac{1}{P_E vN^2}. \end{aligned}$$

### Performance analysis

Below, we employ metrics such as correctness, security, and achievable precision for the performance analysis of the QSM protocol. We also augment a comparative analysis for the intrinsic precision-security trade-off in such experiments.

#### Correctness

In this subsection, we detail the correctness of our protocol. Alice and Bob securely share the Bell state of the form (4) in the first two steps of the protocol. Bob performs the sequential encoding by applying $U_E^N$ on his particle $q^i_B$ with probability $P_E$. This will introduce the phase on the Bell state,

6 $$\begin{aligned} \left| \Psi _{AB} \right\rangle = U_{\phi }^N \left| \Phi _{AB} \right\rangle =\frac{1}{\sqrt{2}} \left( \left| 0_A0_B \right\rangle + e^{\iota N \phi } \left| 1_A1_B \right\rangle \right) , \end{aligned}$$

where

$$\begin{aligned} U_{\phi }^N = \left( U_I \otimes U_E \right) ^{N}. \end{aligned}$$

In the last step, Alice measures the sequentially encoded states using the Bell basis and estimates the parameter. We provide the proof of precision bound (5) in the precision section.

#### Security

In our security analysis section, we consider two scenarios: (1) the adversary attacks the protocol during the transmission of the probe state particle from Alice to Bob, (2) the adversary attacks the protocol when the probe state particle travels back to Alice from Bob.

In the first scenario, the adversary tries to capture the incoming probe state particle and sends a part of her custom-made probe state particle to Bob. After Bob’s encoding, when the probe state particle travels back to Alice, Eve can easily get the parameter value and encode the same parameter into the original probe state. This way, Eve can get the parameter value without being detected. Previously proposed protocol was vulnerable to this kind of attack^10^. However, during the verification steps, this attack will be detected. Bob will select probe state particles with the probability $\left( 1 - P_E \right) /2$ from the sequence $S_B$ and check the correlation with the Alice particles. If Eve performs such an attack, then the correlation between the measurement outcomes of Alice and Bob does not hold, which will abort the protocol.

In the second scenario, the adversary attacks the protocol when the encoded probe state particle travels back to Alice. The encoding is performed locally on the probe state particle. However, this encoding has a non-local effect and requires the whole probe state to decode the parameter. Eve has access to the only probe state particle that is similar to mixed state $Tr_A\{\left| \Psi _{AB} \right\rangle \left\langle \Psi _{AB} \right| \} = I/2$ and can not get any information from this mixed state. The only option left for Eve is to disturb the protocol by encoding a random parameter. Eve can apply the unitary $U_C$

$$\begin{aligned} \left| \Psi '_{AB} \right\rangle = U_C \left| \Psi _{AB} \right\rangle = \frac{1}{\sqrt{2}}(\left| 0 \right\rangle _A \left| 0 \right\rangle _B + e^{\iota N \phi + \beta } \left| 1 \right\rangle _A \left| 1 \right\rangle _B), \end{aligned}$$

to the encoded probe state particle going towards Alice, which will compromise the encoded parameter. However, the parties involved will detect such an attack in our protocol. Bob encodes the parameter on the probe state with probability $\left( 1 - P_E \right) /2$. Eve can not differentiate between encoded and decoy probe states in advance. When Bob applies the random unitary operation $U_C$ on the decoy probe state, the decoy probe state evolves as follows

$$\begin{aligned} \left| \Phi '_{AB} \right\rangle = {U}_C \left| \Phi _{AB} \right\rangle = \frac{1}{\sqrt{2}}(\left| 0 \right\rangle _A \left| 0 \right\rangle _B + e^{\iota \beta } \left| 1 \right\rangle _A \left| 1 \right\rangle _B). \end{aligned}$$

Bob announces the location of these decoy probe states, and Alice performs the Bell state measurement (BSM) and computes the error rate. If she finds errors in these decoy probe states, she will abort the protocol; otherwise, she will continue to the next step.

In both scenarios mentioned above, we can easily calculate the probability of detecting Eve $P_d$. For every correlation check, the probability that Eve escapes undetected is 1/2, similar to the E91 protocol^30^. As previously mentioned, the parties can abandon the protocol if they detect Eve during the transmission of the probe state (from Alice to Bob or from Bob to Alice). The probability of the state being a decoy is $(1-P_E)/{2}$, and the number of probe states intercepted by Eve is *l*. Then, the probability of detecting Eve for one side transmission of the probe state will be

7 $$\begin{aligned} P_D = 1-(\frac{3+P_E}{4})^l. \end{aligned}$$

**Figure 2:**
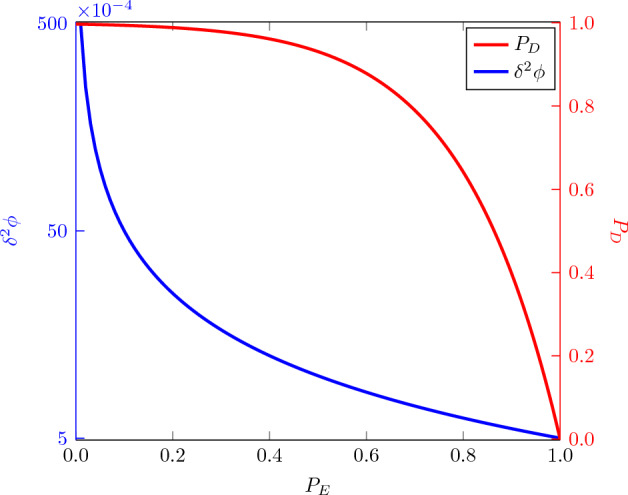
Probability of detecting Eve $P_D$ and variance of estimand parameter $\delta ^2 \phi$ compared with encoding probability $P_E$. Increasing $P_E$ results in increased precision, but at the expense of security of the protocol.

As *l* increases, $P_D$ increases until it reaches unity in the asymptotic limit of *l*, as depicted in Fig. 2.

#### Precision

After validating the channel’s security, Alice receives the quantum state (6) and performs the parameter estimation process. The QFI attainable in this scenario is directly related to $P_E$, which shows the number of resources employed for the parameter estimation. She measures the Bell observable $B= \left| 0 \right\rangle \left\langle 1 \right| ^{\otimes 2}+\left| 1 \right\rangle \left\langle 0 \right| ^{\otimes 2}$. The expected value of the observable is

$$\begin{aligned} \langle B \rangle = \left\langle \Psi _{AB} \right| B\left| \Psi _{AB} \right\rangle = \cos (\phi ), \end{aligned}$$

whereas the variance of the observable becomes

$$\begin{aligned} \delta ^2 B = \langle B^2 \rangle - \langle B \rangle ^2 = \sin (\phi ). \end{aligned}$$

The results of the measurements are subjected to classical post-processing, and Alice gets an estimate ${\hat{\phi }}$. For *v* repetitions of the protocol, the number of resources used for parameter estimation becomes $v' = P_E v$. We can substitute this value with expectation and variance of the observable in (3) to evaluate $\delta ^2 \phi$ in (5).

#### Precision-security trade-off

Under the finite resource assumption, there is an intrinsic trade-off between achievable precision and security. The particles not utilized for parameter encoding are actually employed as decoy states. We can quantify this trade-off provided the precision does not exceed the HL and is equal to or less than the SQL to ensure a quantum advantage, which leads to

8 $$\begin{aligned} \frac{1}{N} < P_E \le 1. \end{aligned}$$

Figure 2 gives a detailed description of the precision-security trade-off relationship. As $P_E$ increases, the number of resources delegated for the parameter estimation process increase, and thus the variance of the estimate decreases. As a consequence, the security of the protocol suffers since fewer resources are remained for the security checking phase. The values of $P_E$ for $P_E > 1/N$ would ensure a quantum advantage. Application-specific performance goals of secure quantum metrological scenarios may vary; conventionally, we can sacrifice one aspect at the expense of the other or analyze the trade-off to get the benefits of both.

## Discussion

We have proposed a quantum secure metrology protocol that enables the secure estimation of a physical parameter by a remote party. The protocol provides a precision scaling beyond the SQL. We proved its robustness against multiple attacks in the security analysis. The controlled encoding strategy ensures the secrecy of the parameter. The allocation of quantum resources in terms of encoded and decoy states enables the detection of malicious parties in the channel. The resource allocation causes a precision security trade-off which we analyzed numerically and provided a bound on the allowable range of $P_E$ values. Due to the inherent simplicity of the process, we can extend the protocol to multiple parties. Alice shares Bell states with *K* parties in such a setting, creating a distributed sensing network. Each participant $B_i \in \{B_1, B_2,.......,B_K\}$ encodes their particle with the local parameter $\phi _j \in \{\phi _1,\phi _2,.......,\phi _K\}$ and returns it to Alice, who performs multi-parameter estimation. Such topologies are extremely cost-effective as we can delegate the task of data processing to a single node in the network. Cluster sensing networks employing classical sensing nodes frequently use such schemes for applications such as spectroscopy and magnetometry^31,32^.

In practical implementations of QSM, the probe would have to pass through an imperfect quantum channel separating the source and the system. Our protocol allows for the global measurement of the state, which is crucial for gaining optimal results when employing entangled probes^33–35^. Performing these measurements is also practical, as recent works have proposed experimental implementations of deterministic Bell state analyzers^36,37^. However, generating deterministic *N*-partite GHZ state analyzers remains a challenge^35^. Thus, the practical performance of QSM protocols employing multi-partite probes will suffer not only the unfavorable generation and distribution of entanglement but also the non-realizable measurement techniques. Furthermore, we can observe the specific case proposed in our protocol as synonymous with the ancilla-assisted quantum metrology scheme, which yields higher QFI than the multi-partite case in the high noise regime^34,38^. Recent advances in quantum memories coupled with the relatively simple nature of biparite entanglement has also enabled efficient preservation of two spatially separated quantum particles^39–41^. These developments are vital in realizing practical QSM as the eavesdropping check during such protocols require the entangled states to retain a high degree of fidelity. We can also incorporate quantum repeaters in our protocol to enable state transfer among relatively distant nodes for large-scale sensing networks^42–45^. This idea is further aided by the advances in device-independent quantum key distribution as well as experimental realizations of high-fidelity long-distance secure quantum communication^46,47^. Furthermore, it is relatively trivial to perform entanglement purification and error correction to reduce the effect of noise in the channel when employing Bell states. For instance, the conception has also been utilized for creating interferometric telescopes^48^. Other issues in practical networks are side-channel attacks due to the imperfect isolation of private spaces possessed by both parties, Alice and Bob. This issue can be resolved by adopting alternate methods wherein virtual channels replace real channels, in turn, effectively culminating unwanted probing of private spaces held by the network recipients^49^.

Our work aims to accelerate the near-term deployment of QSM networks. Future works include characterizing its security and metrological performance in open dynamical systems with various noise models and eavesdropping attacks. Thus, the QSM formalism will create further opportunities for applications regarding the integrated paradigm of network security and network sensing.

## Data Availability

The datasets used and analyzed during the current study are available from the corresponding author upon reasonable request.
